# Prevalence and clinical features of anxiety disorders: Tunisian study about 436 subjects

**DOI:** 10.1192/j.eurpsy.2022.992

**Published:** 2022-09-01

**Authors:** M. Jabeur, L. Gassab, A. Ayadi, B. Ben Mohamed, F. Zaafrane, L. Gaha

**Affiliations:** Research laboratory LR 05 ES 10 “Vulnerability to Psychotic Disorders”, Faculty of medicine, University of Monastir, Psychiatry Department, University Hospital Of Monastir, Monastir, Tunisia

**Keywords:** Prevalence, Anxiety disorders, incidence, Tunisian trial

## Abstract

**Introduction:**

Anxiety disorders represent one of the most common mental disorders following a chronic course.

**Objectives:**

The aim of our study is to determine the prevalence, incidence and clinical characteristics of these disorders.

**Methods:**

We conducted a retrospective and descriptive study about 436 outpatients fulfilling the DSM-V diagnostic criteria for anxiety disorder and followed in the department of psychiatry of Monastir (Tunisia) between 1998 and 2017. Selective mutism and separation anxiety were excluded for lack of cases.

**Results:**

The overall prevalence was 5.51%. Panic Disorder was the most prevalent anxiety disorder subtype (3.2%). The incidence of anxiety disorders in the last years has increased from 3.31% in 1998 to 7.5% in 2017. The mean age at diagnosis was 37.76±12.87 years [16-77]. Female gender was the most prevalent in overall anxiety disorders with a sex ratio of 0.78, however, a significant male predominance was recorded in Social Anxiety Disorder (SAD) with a sex ratio of 1.85. Generalized Anxiety Disorder patients were more likely to have low educational level (OR= 1.879), to be laborers (OR=2.55), to be married (OR=2.418) and to have children (OR=2.564) whereas SAD patients were more likely to have higher education (OR=9.118), to be students (OR=5.565), to be single (OR=11.325) and have no children (OR=7.464).

**Conclusions:**

This study highlignts the frequency of anxiety disorders and the fact that being a middle-age married woman with kids make oneself more prone to have an anxiety disorder. Specific attention should be paid to these anxiety disorders with early preventive programs.

**Disclosure:**

No significant relationships.

